# Using RNA-seq data to select reference genes for normalizing gene expression in apple roots

**DOI:** 10.1371/journal.pone.0185288

**Published:** 2017-09-21

**Authors:** Zhe Zhou, Peihua Cong, Yi Tian, Yanmin Zhu

**Affiliations:** 1 Institute of Pomology, Chinese Academy of Agricultural Sciences, Xingcheng, Liaoning, P. R. China; 2 United States Department of Agriculture, Agricultural Research Service, Tree Fruit Research Laboratory, Wenatchee, Washington, United States of America; Huazhong Agriculture University, CHINA

## Abstract

Gene expression in apple roots in response to various stress conditions is a less-explored research subject. Reliable reference genes for normalizing quantitative gene expression data have not been carefully investigated. In this study, the suitability of a set of 15 apple genes were evaluated for their potential use as reliable reference genes. These genes were selected based on their low variance of gene expression in apple root tissues from a recent RNA-seq data set, and a few previously reported apple reference genes for other tissue types. Four methods, Delta Ct, geNorm, NormFinder and BestKeeper, were used to evaluate their stability in apple root tissues of various genotypes and under different experimental conditions. A small panel of stably expressed genes, MDP0000095375, MDP0000147424, MDP0000233640, MDP0000326399 and MDP0000173025 were recommended for normalizing quantitative gene expression data in apple roots under various abiotic or biotic stresses. When the most stable and least stable reference genes were used for data normalization, significant differences were observed on the expression patterns of two target genes, *MdLecRLK5* (MDP0000228426, a gene encoding a lectin receptor like kinase) and *MdMAPK3* (MDP0000187103, a gene encoding a mitogen-activated protein kinase). Our data also indicated that for those carefully validated reference genes, a single reference gene is sufficient for reliable normalization of the quantitative gene expression. Depending on the experimental conditions, the most suitable reference genes can be specific to the sample of interest for more reliable RT-qPCR data normalization.

## Introduction

Gene expression analysis is an important aspect for inferring the function of a gene of interest. Reverse transcription quantitative PCR (RT-qPCR) is a widely-applied technique for gene expression analysis for its the ability to detect a wide-range of gene expression with dynamics and accurate quantification [1–4]. However, the accurate and repeatable RT-qPCR data depend on careful data normalization using reliable reference genes [5, 6]. Data normalization is crucial for minimizing the impacts of technical noise such as the amount of starting materials, RNA integrity, reverse transcription efficiency and cDNA sample loading variation. The ideal reference genes should exhibit low variation in expression across diverse sample types and/or experimental conditions [7, 8].

Before the availability of large genomic data sets, reference genes have been traditionally selected from those genes involved in “housekeeping” cellular functions, such as ubiquitin-conjugating enzyme (*UBC*), polyubiquitin (*UBQ*), β-actin, α- and β-tubulin, and glyceraldehyde 3-phosphate dehydrogenase (*GAPDH*). However, these genes have been shown to exhibit surprisingly high expression variance among tissue types and/or under different conditions [9–14]. The best suitable reference genes have been shown to be variable in different tissues or under specific experimental conditions within a species [15–19]. Therefore, the importance of validating reference gene expression stability in the samples of interest is likely more important than previously realized. It has been suggested that the term ‘reference genes’ should apply to only those genes whose stable expressions have been experimentally validated in the respective tissues for a species, or under the given set of experimental conditions [17].

Large-scale and high throughput RNA sequencing (RNA-seq) technology has become a mainstay of global gene expression analysis. With its enormous capacity, RNA-seq and associated bioinformatic software allow simultaneously identification and quantification of the entire inventory of a transcriptome in a tissue under specific conditions [20–24]. The primary utilization of RNA-seq experiments are designed for identifying differentially expressed genes under certain conditions. The genes that exhibit least variable expression levels across tissue types and/or conditions can be exploited as the source of good candidates for potential reference genes [25, 26]. Several methods have been developed for analyzing the expression stability of candidate reference genes among RT-qPCR data including geNorm, NormFinder and BestKeeper [27–29].

Apple (*Malus* × *domestica* Borkh.), a member of the Rosaceae family and Pyroideae subfamily, is one of the most popular perennial tree fruits. Most of the research interests have been focusing on the fruit, leaf and flower. Apple roots and their responses to various stress conditions are less-explored. The apple reference genes, which are specifically suitable for studying quantitative gene expression in apple root, have not been carefully analyzed [26, 30]. In this study, a set of candidate genes with low expression variance were selected from a large data set of RNA-seq based apple root transcriptome analysis. The suitability of these candidates for using as RT-qPCR reference genes was evaluated in apple root tissues from different genotypes and under various experimental conditions. A traditional “housekeeping” gene for encoding actin in apples and four previously reported reference genes, which were previously analyzed in apple fruit tissues [30], were also evaluated. The objective of this study is to carefully validate a panel of reliable reference genes for RT-qPCR expression analysis in apple roots under various biotic and abiotic stresses.

## Materials and methods

### Preparation and maintenance of apple rootstock plants by tissue culture procedure

Tissue culture based micropropagation procedures were used to obtain cloned plants for different apple rootstock genotypes as described previously [31]. To minimize the influence of nongenetic factors, a synchronized micropropagation procedure was employed to generate plants of equivalent developmental stage for the genotypes of apple rootstock used in this study. Specifically, the root tissues of 4-week-old plants after root induction in tissue culture medium were transferred to pasteurized (in an oven at 85°C for two consecutive overnights) Sunshine^™^ potting mix soil (SUN GRO Horticulture Ltd, Bellevue, WA) for a period of one week with “in-soil” acclimation in a growth chamber for further differentiation of root tissues. For treatment of abiotic stress conditions, autoclaved construction sands were used instead of potting mix soil. To reduce the transplanting effects from tissue culture medium to soil condition, a transparent 7′′ Vented Humidity Dome (Greenhouse Megastore, Danville, IL) was placed on top of a 10 × 20-inch flat tray holding the pots for retention of humidity. Root tissues were collected by flash freezing in liquid nitrogen, and then stored at −80°C before RNA isolation. Plant materials used in this experiment are from an elite apple rootstock cross population between ‘Ottawa 3’ x ‘Robusta 5’. Many agronomical traits such as dwarfness, cold hardness and disease resistance are known to be segregated among the progeny from this cross population. The numbers used for describing the plant genotypes, such as #58, #75 and #115, represent the individual progeny from this cross population.

### Total RNA isolation and high-throughput mRNA sequencing

Total RNA isolation followed the method previously described in Zhu et al. [31]. Root tissues of B.9 and G.935 were represented by three biological replicates, and each replicate included the pooled root tissues from six plants. The frozen root tissue samples were ground to a fine powder in liquid nitrogen. RNA quantity was determined using a Nanodrop spectrophotometer (ND-1000; Thermo Fisher Scientific). The RNA integrity number (RIN) was evaluated using an Agilent 2100 Bioanalyzer. Only RNA with an RIN value of ≥ 8 was used for RNA-seq. Oligo (dT) magnetic beads were utilized to isolate poly-(A) tails containing mRNAs from total RNA and then fragmentation buffer was added to break mRNA to short fragments. Using these short fragments as templates, first-strand cDNA was synthesized using reverse transcriptase and a random hexamer primer. Second-strand cDNA fragments were then synthesized using a buffer, DNA polymerase I, dNTPs, and RNase H. After purification and paired-end (PE) repair protocols were performed, the cDNA fragments were ligated to sequencing adapters and amplified using PCR to obtain the final PE cDNA library. The library preparation and RNA-sequencing with 150 bp paired-end (PE) were completed at the Center for Genome Research and Biocomputing in Oregon State University using an Illumina HiSeq^™^ 3000 (Illumina Inc., San Diego, CA, USA).

### Mapping of sequence reads

Reads from B.9 (susceptible) and G.935 (resistant) libraries were mapped to the nucleotide sequences of predicted coding genes of the apple (*Malus* x *domestica*) genome sequences v3.0.a1 (https://www.rosaceae.org/analysis/162) using the ultrafast, memory-efficient short read aligner Bowtie 2–2.2.5 which utilizes a Burrows-Wheeler index [32]. Count data were obtained for each coding sequence. Estimation and statistical analysis of expression levels using count data of each gene with 3 replicates for each library was performed using the DEseq2 package [33] and R x64 3.3.1 program (https://www.r-project.org/). The annotation of these genes was carried out by BLASTP [34] against NR (nonredundant protein sequences) database and a BLAST database containing genomic sequences for Arabidopsis (Arabidopsis thaliana), corn (*Zea mays*), Medicago truncatula, rice (*Oryza sativa*), and tomato (*Solanum lycopersicum*).

### Selection of candidate reference genes and primer design

Selection of candidate reference genes from in-house RNA-seq data followed the criteria below: 1. The coefficient variance (cv) values were smaller than 0.3; 2. The calculated Log_2_FC (fold change) values at each time point were between -0.1 to +0.1; 3. The basemean values were 500–3,000. The nucleotide sequence of each candidate gene was downloaded from the Genome Database for Rosaceae (GDR, http://www.rosaceae.org/). Primers were designed using the Primer Quest tool (Integrated DNA Technologies) with the following criteria: GC content 45–65%, Tm >50°C, primer length 20–24 bp, and amplicon size 150–200 bp.

### Abiotic stress treatments

After 4 weeks of culture and 1 week of “in-soil” acclimation, ‘Gala’ plants were subjected to four different abiotic stresses. For salinity and drought treatments, plants were irrigated with different stresses in solutions containing 1/2 strength Murashige and Skoog (MS) medium at pH5.6 as follows: salinity (200 mM NaCl) and drought (15% PEG 6000); the control plants were irrigated with 1/2 MS only. To simulate cold stress, the1/2 MS medium watered plants were placed at 4°C in light. For nutrition shortage treatment, plants were only irrigated with water, whereas the control plants were irrigated with 1/2 strength of MS medium. The roots of all treated plants from three biological replicates were carefully harvested after 2 days, immediately frozen in liquid nitrogen, and then stored at −80°C until total RNA extraction.

### Inoculum preparation, inoculation and root tissue collection

Inoculum of *P*. *ultimum* was prepared as previously described [35]. The inoculation of seedlings with *P*. *ultimum* was performed by dipping the root system into the inoculum solution of 2 x 10^3^ CFU (colony forming unit) for 5 s and then planting treated seedlings into pasteurized Sunshine^™^ potting mix and water thoroughly. Control plants were mock inoculated with 0.5% methyl cellulose solution and then transplanted and maintained the same manner as pathogen infected plants. Inoculum of *R*. *solani* AG-5 strain 1007 were prepared by growing *R*. *solani* in 500 mL Erlenmeyer flasks containing 250 mL of oat bran and 80 ml of distilled water. The flasks were sterilized by autoclaving for 90 minutes on two consecutive days and inoculated with a culture of *R*. *solani* growing on 1/5-strength PDA. Flasks were incubated at 20°C for 14 days, and then inoculum was air-dried in a laminar flow hood. Ground oat grain inoculum of *R*. *solani* AG-5 were homogeneously mixed into soil at a rate of 0.25% (wt/wt), and soils were planted after 24 h of incubation at 20 to 23°C [36]. The pathogen inoculated and mock inoculated plants were maintained in an environmental growth chamber at 23°C and 95% humidity, under a 12/12 h light/dark photoperiod. Plants root tissues were harvested at 0, 24, 48 and 72 hpi. Root tissues were collected by excavating from soil, washing with water and flash-freezing in liquid nitrogen. Root tissues of six plants were collected and pooled as a biological replicate at each time point per treatment. For concise description of root samples from specific apple rootstock genotype and pathogen type, a short name was used, such as “#58 Pu” for #58 germplasm line of apple rootstock genotype infected by *P*. *ultimum*, or “115 Rs” for #115 germplasm line infected by *Rhizoctonia solani*.

### RNA isolation, cDNA synthesis and RT-qPCR

Total RNA isolation and cDNA synthesis were as previously described [37]. The cDNA was diluted 20 times and 0.6 μL aliquot was used in a 15 μL quantitative PCR (qPCR) reaction mix: 0.45 μL SYBR Green I dye (Invitrogen, Grand Island, NY), 1x iTaq buffer (Biorad, Hercules, CA), 0.2 mM dNTP (Applied Biosystems, Waltham, MA), 2.5 mM MgCl_2_, 0.3 units of iTaq DNA polymerase (Biorad, Hercules, CA), and 0.2 μM forward/reverse primer (IDT, Coralville, IA). RT-qPCR was performed in 96-well plates by using an iQ5 real time qPCR detection system (Biorad Lab, Hercules, CA) and the following protocol: cycle conditions of 3 min at 95°C and 40 cycles of 10 s at 95°C and 30 s at 59°C. The melting curve for each amplicon was obtained from 60° to 95°C to verify primer specificity. All assays were carried out in three technical and biological replicates with template-free negative controls being performed in parallel. PCR efficiency and correlation coefficient (R^2^) for each primer set was calculated by the slopes of standard curves generated in Microsoft Excel 2016 from a 5-fold cDNA dilution series (1:5, 1:25, 1:125, 1:625 and 1:3125).

### Statistical analysis of gene expression stability

For evaluating gene expression stabilities, four different methods were used: ΔCt [38], geNorm [28], NormFinder [27] and BestKeeper [29]. The comprehensive ranking of the stability of the reference genes was determined using RefFinder [39]. Raw RT-qPCR data were obtained using iQ5 Optical System Software V2.1, and the cycle threshold (Ct) values were used to analyze the expression levels of candidate reference genes. For delta-Ct (ΔCt) method, raw Ct values were transformed to relative quantities using the delta-Ct formula Q = E^ΔCt^ where E is the efficiency of the gene and ΔCt equals the lowest Ct value from the data set minus the Ct value of the sample in question. The raw Ct values can be directly used for the BestKeeper, whereas genNorm and NormFinder employ relative quantity to compare gene expression stability. For geNorm, the transcriptional stability of the candidate genes is ranked by the M value, which is calculated based on the average pairwise variation of each gene with other candidate genes [28]. Genes with lower M values are considered transcriptionally more stable under tested conditions than genes with higher M values. In addition, geNorm calculates the pairwise variation (V value) as a guide to determine the optimal number of reference genes required for target gene expression normalization. It is not necessary to add third reference gene if the V value is below or equal to 0.15. NormFinder, an ANOVA based model, takes inter- and intra-group variation into account to calculate the expression stability value [27]. In this analysis, lower variation values indicate more stable expression patterns. BestKeeper ranks the candidates’ stability by three parameters: the standard deviation (Std dev), coefficient of variation (CV) and correlation coefficient (r) [29]. The most stable reference gene is the one with the smallest CV and Std dev value. Finally, RefFinder, a web-based analysis tool, was used to integrate the results from delta-Ct methods and the three programs to get the overall ranking [40].

### Validation of reference gene analysis

To validate the reliability of the evaluated reference genes, two genes, *MdLecRLK5* (MDP0000228426) and *MdMAPK3* (MDP0000187103), which showed relatively high expression levels in roots after pathogen inoculation in our transcriptome sequencing database, were used as target genes. Two sample types of “#58 Pu” and “#115 Rs” were used for expression analysis for these two target genes. RNA extraction and cDNA synthesis were performed as mentioned above. The expression levels of *MdLecRLK5* and *MdMAPK3* in “#58 Pu” tissues were normalized using the most stably expressed reference gene (MDP0000095375), the 2nd most stably expressed reference gene (MDP0000147424), the combination of two most stably expressed reference genes (MDP0000095375 + MDP0000147424) and the least stably expressed gene (MDP0000245145). Similarly, the expression of two target genes in “#115 Rs” samples, the four sets of reference gene combinations were MDP0000095375, MDP0000336547, MDP0000095375 + MDP0000336547, and MDP0000245145. The *MDP0000211280*’s expression level in 115 after *R*. *solani* inoculation was too low to be used for normalization, so the 2nd least stably expressed gene MDP0000245145 was selected instead. The primer pairs for RT-qPCR for two target genes were: 5’- GAATGCCAGCTTCAGAGTTAGA-3’ (forward) and 5’- TCCGATCCCTTTCTCTTGTTTC-3’ (reverse) for *MdLecRLK5*, 5’- GAGAAGAAAGTCCTCCACCAAA-3’ (forward) and 5’- GTGGCGTCGAACATCAAATTC-3’ (reverse) for *MdMAPK3*. The target genes’ relative expression levels were calculated using the 2^−ΔΔCT^ method (the comparative Ct method) [41]. Data were then processed with the software SPSS V19.0 for Windows (SPSS, Chicago, IL, USA) to perform a one-way analysis of variance (ANOVA) test and multiple comparisons.

## Results and discussion

### Selection of candidate genes from a comprehensive apple root transcriptome data set

A panel of 15 candidate reference genes were selected for validating their expression stability and suitability as reliable reference genes for normalizing RT-qPCR data in apple root tissues under various biotic and/or abiotic stresses (Table 1). The first ten genes in Table 1 were selected from a recent RNA-seq dataset, where their low variance of expression patterns were determined between two apple rootstock genotypes, with or without pathogenic stress and at different time points after inoculation. Several criteria were applied to select candidate genes with low variance between treatments and intermedium expression level in apple roots: 1. The calculated coefficient variance ≤ 0.3; 2. The Log_2_FC values of paired-wise comparison were between -0.1 to +0.1; 3. The basemean values (a measurement of normalized numbers of mapped reads) from RNA-seq data set were about 500–3,000 to exclude those with extremely highly or lowly-expressed genes. Four reported apple reference genes [30], which were primarily tested in various fruit tissues were also included in this study. An actin gene (MDP0000752428), which has been previously selected based on its “house-keeping” function and with limited validation [35], was also included in this study. The primer sequences and their property during PCR reaction were shown in Table 2.

**Table 1:** Evaluation of selected reference genes using RNA-Seq data.

MDP #	Basemean	CV	SD	Log_2_Foldchange value					
				Bc1vsBp1	Bc2vsBp2	Bc3vsBp3	Gc1vsGp1	Gc2vsGp2	Gc2vsGp3
MDP0000121104	1430.29	0.2063	295.14	0.086017	-0.09139	0.048657	0.012115	-0.0265	-0.07451
MDP0000147424	759.55	0.2332	177.20	0.021128	-0.04327	-0.08832	-0.09394	-0.06962	-0.02599
MDP0000200579	1521.81	0.1879	285.95	0.060406	-0.08321	0.053851	-0.08034	-0.03147	-0.00264
MDP0000211280	873.19	0.2423	211.62	-0.00103	-0.0068	0.062149	-0.09355	0.091459	-0.01017
MDP0000233640	1426.34	0.2178	310.77	-0.00034	-0.07927	0.013842	0.064146	-0.00572	0.007154
MDP0000245145	1909.57	0.2389	456.26	0.047466	0.070705	0.019014	-0.03362	0.085218	0.061582
MDP0000314861	1145.06	0.1988	227.65	0.025069	0.060268	0.006978	0.00763	0.048521	0.000123
MDP0000315565	607.26	0.2379	144.47	0.062952	-0.01437	0.071055	0.032791	-0.02737	-0.04487
MDP0000831483	1900.44	0.2815	534.90	0.04719	0.012713	-0.05155	-0.0221	-0.09805	-0.00216
MDP0000913709	1526.18	0.2427	370.41	0.020355	-0.06111	0.007801	-0.07658	-0.09141	0.007545
MDP0000095375	1354.08	0.1960	265.48	-0.12277	-0.24552	-0.07419	0.012061	-0.22095	-0.02428
MDP0000173025	977.40	0.2685	262.47	0.228833	0.304735	0.152197	0.0494	0.193024	0.270897
MDP0000326399	827.90	0.2105	174.31	0.010125	0.06489	0.053718	0.11714	-0.10193	-0.10296
MDP0000336547	388.34	0.2521	97.93	0.162455	-0.01964	0.193567	-0.01862	-0.36356	0.127541
MDP0000752428	1865.52	0.2666	497.42	-0.05087	-0.25225	0.013893	-0.13111	0.263177	-0.08603

MDP: predicted gene model from the apple (*Malus* x *domestica*) genome sequences v3.0.a1 (https://www.rosaceae.org/analysis/162). CV: coefficient of variation, SD: standard deviation. Log_2_fold change: normalized values of transcript abundance between mock inoculation control and *Pythium ultimum* infected apple root at different time point for two genotypes. Bc, B.9 roots mock inoculated as control, Bp, B.9 roots infected by *P*. *ultimum*; Gc, G.935 roots mock inoculation as control, Gp, G.935 roots infected by *P*. *ultimum*. Number 1, 2, 3 represent 1, 2 and 3 day(s) after inoculation. The highlighted genes listed at the bottom of the table are from previous report (30, 31) but not carefully validated in apple root tissues. RNA-seq data were deposited in GEO (Gene Expression Omnibus) (www.ncbi.nlm.nih.gov/geo/query/acc.cgi?acc=GSE62103) with the accession number of GSE62103.

**Table 2:** Description of candidate reference genes and primer sequences for qRT-PCR.

MDP# Gene description	Primers F/R [5’-3’]	Amplicon size (bp)	Tm (°C)	PCR efficiency (%)	Regression coefficiency (R2)
MDP0000095375 Casein kinase II subunit beta-4 isoformX2	F-5’ ACATCGAGAGCTTTGGCTAAC 3’ R-5’ CTCCATCAGAACCACTGACATC 3’	187	82.0	98.75	0.9993
MDP0000121104 WD repeat-containing protein DWA2	F-5’ GCATTTGATTCGGCTCTCTTC 3’ R-5’ GCTCAGGGATCTGCCATATT 3’	169	84.5	100.83	0.9982
MDP0000147424 Protein GRIP	F-5’ CTCAGGCTGATCGCAATGATA 3’ R-5’ CCAGCTCCTCAATTTCCTCTT 3’	168	84.5	105.35	0.9970
MDP0000173025 Membrane related	F-5’ ATGGAGAGATGGAATGGCAAAG 3’ R-5’ GTGAGCATCGGATCCCATTTAG 3’	193	85.0	92.92	0.9992
MDP0000200579 Ubiquitin-conjugating enzyme E223	F-5’ CCTAATCGTGTGGAAGGTACTG 3’ R-5’ ATGAGGGAAGAAGACACCAATC 3’	192	82.0	100.39	0.9998
MDP0000211280 26S proteasome non-ATPase regulatory subunit 6	F-5’ TCTGTTACCAAGATCGCTGATG 3’ R-5’ ATGGAAGACCAAGTCCATCTTT 3’	179	83.5	97.52	0.9998
MDP0000233640 Ribosome biogenesis protein BMS1	F-5’ CAAGGCCGAGAAGAAGAAGAA 3’ R-5’ TCCATGAACCAGAACGACATAC 3’	198	88.0	101.61	0.9968
MDP0000245145 Poly(U)-specific endoribonuclease-B	F-5’ GGGCCAGTGGAATGGATATAAG 3’ R-5’ CCCTCCACCACAGTCAATTT 3’	189	83.0	101.78	0.9996
MDP0000314861 PRA1family protein A1-like	F-5’ TCTCATCGCAGCTGTTCTTAC 3’ R-5’ GAGGCCGTCCACATATGAAT 3’	196	82.5	97.80	0.9988
MDP0000315565 Trafficking protein particle complex subunit 12	F-5’ GATCGGCTGTACTGCTTGTTAG 3’ R-5’ GGGTCTGTGTAATCACGGTTAAG 3’	193	81.5	108.20	0.9986
MDP0000326399 Glycerol-3-phosphate acyltransferase 3	F-5’ CGTGGAACTGGAATGTGTATCT 3’ R-5’ ACTCTACCAAGAGCCTCTCTAA 3’	195	85.5	94.01	0.9963
MDP0000336547 SGF 29 tudor-like domain-containing protein	F-5’ AGCTACTCCTGAAGTGGTAGAG 3’ R-5’ GCTCATTACCTTCCATCCTTCTT 3’	188	85.0	106.96	0.9994
MDP0000752428 Actin like	F-5’ AGTCCAAGCGTGGTATCTTAAC 3’ R-5’ CTGGGTCATCTTCTCACGATTT 3’	188	87.0	98.97	0.9982
MDP0000831483 Transcription factor GTE8-like isoformX1	F-5’ GGACTCTGGTGAGGATGAGATC 3’ R-5’ CCGGGTCATTCCCTTTACAT 3’	199	80.5	104.17	0.9977
MDP0000913709 Ubiquitincarboxyl-terminal hydrolase isozymeL5-like	F-5’ GCAGAAGGTAGCTGACAAAGA 3’ R-5’ TCGATGCGTTCCTGGATTAC 3’	182	85.0	102.25	0.9997

MDP: predicted gene model from the apple (*Malus* x *domestica*) genome sequences v3.0.a1 (https://www.rosaceae.org/analysis/162). Gene description is based the annotation by BlastP against NR (nonredundant protein sequences) database.

The transcriptome dataset, which was used to select these genes with low variance of transcript levels, was generated with most recent sequencing format of paired-end reads of 150-bp by Illumina HiSeq 3000 platform. Based on normalized values of mapped reads and calculated gene expression levels, about one third of the gene models predicted in current apple genome assembly were expressed in root tissues. This data set encompassed two apple rootstock genotypes (a resistant G.935 and a susceptible B.9), two treatments (infected by soilborne necrotrophic pathogen *Pythium ultimum* or mock-inoculated control), three biological replicates for each sample and root tissues collected at four time-points (0, 24, 48 and 72) hour post inoculation. Therefore, from this carefully designed RNA-seq experiment, the expression values for each gene were represented by 42 raw data points in term of normalized values of mapped reads.

### Expression levels, primer specificity and amplification efficiency

**Fig 1:**
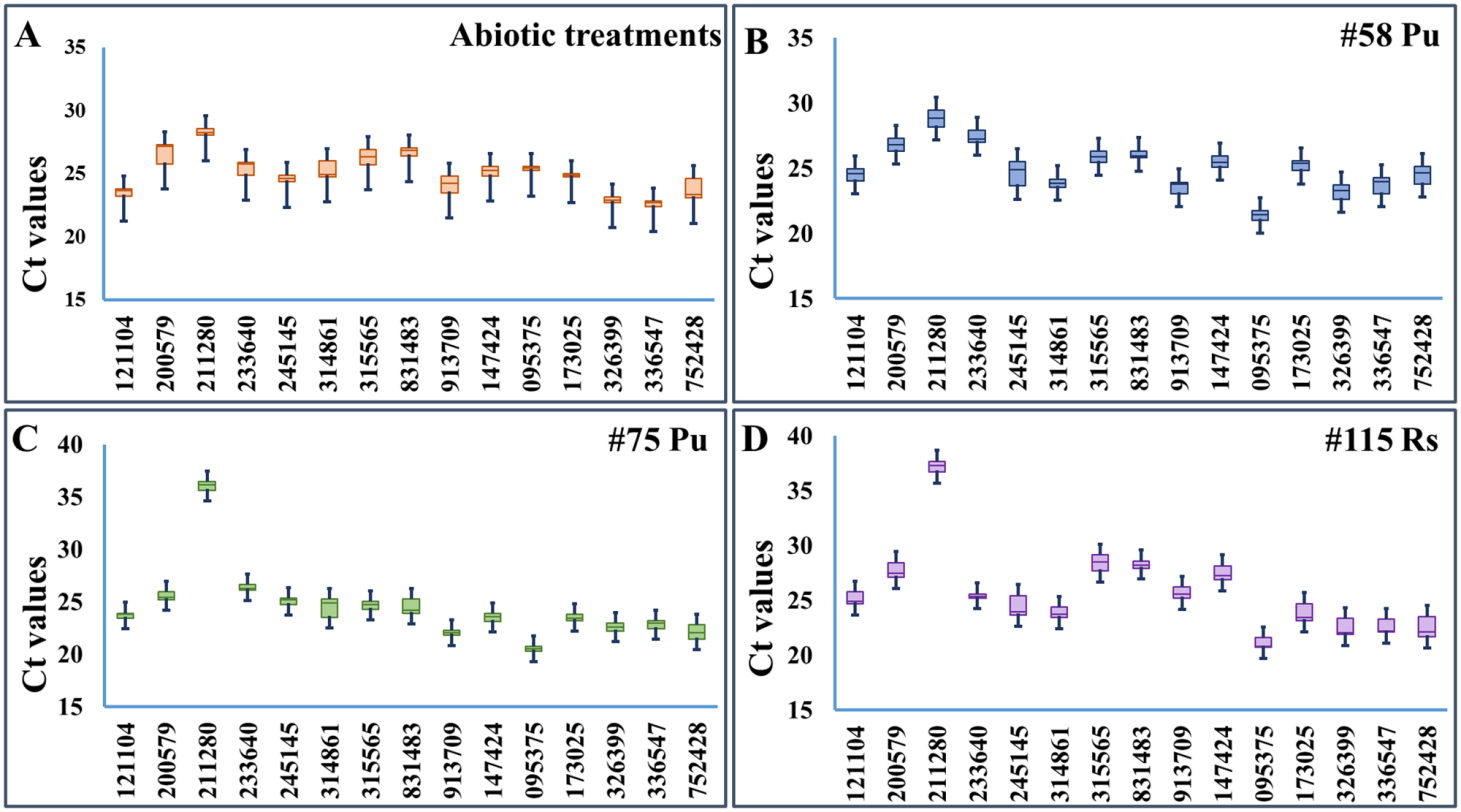
Cycle threshold (Ct) values of the fifteen tested genes across all samples. (A) Abiotic stresses treated root tissues of cultivar ‘Gala’; (B) *P*. *ultimum* infected #58 roots; (C) *P*. *ultimum* infected #75 roots; (D) *R*. *solani* infected #115 roots. For each box, the upper and lower edges indicate the 25th and 75th percentiles, while whisker caps represent the maximum and minimum values. The line across the box depicts the median.

Amplicon specificity and PCR efficiency for the selected candidate genes were tested using root tissues from several apple rootstock genotypes under various treatments. Single PCR amplicon for each primer set was confirmed by melting curve analysis with Tm values ranging from 80 to 88°C and amplification efficiency of 92–108% (Table 2). The PCR efficiency and the R^2^ are used for measuring how well the data fit the standard curve, which reflects the linearity of the standard curve and the inhibition of PCR. Since all tested reference genes had over 90% but less than 110% efficiency and over 0.99 R^2^, they were generally considered suitable for further RT-qPCR analysis [42]. The detected Ct values ranged from 21 to 28 for 14 out of 15 selected genes, and the median Ct values for majority of the selected candidate reference genes were between 22 to 25 (Fig 1). The observed medium expression level could partly be attributed to the selection criteria of basemean values of 500–3,000. The basemean value is a more modern method of normalized quantification of mapped reads to a gene in RNA-seq data set, and the criteria of 500–3,000 was intended to exclude those with extreme expression intensity in apple roots.

The variation ranges of Ct values appear to be specific to sample types for the 15 reference genes (lower values represent higher stability). For abiotic treatments, MDP0000245145 had the lowest value (1.04 cycles), whereas MDP0000200579 had the highest (3.78 cycles). For *P*. *ultimum* infected #75 and #58, MDP0000173025 and MDP0000831483 showed the least variation (1.03 cycles and 1.25 cycles), while MDP0000211280 and MDP0000314861 showed the most (4.41 cycles and 5.04 cycles). For *R*. *solani* infected #115, MDP0000233640 had the narrowest range of variation (1.17 cycles), while MDP0000245145 had the widest (3.55 cycles). Notably, the detected Ct values for a few selected genes were not proportionally coordinated with basemean values. One obvious outlier is MDP0000211280, whose expression levels showed unexpected high Ct values. The higher than expected Ct values are more noticeable in the tissue types of “115 Rs” and “#75 Pu” tissues. Both lines (#115 and #75) are known to be susceptible to these pathogens (personal communication). This observation may indicate its expression was suppressed in selected genotypes during pathogenesis. Therefore, MDP0000211280 is less likely to be a stably expressed gene under pathogenic pressure in some genotypes. The discrepancy between basemean values and Ct values could also derive from the different algorithms between RNA-seq read mapping and the dynamics of PCR amplification. For example, the large size of gene family and wide-spread genome rearrangement of apple genome could lead to the ambiguity of accurately mapping the reads to a specific gene family member [43]. On the other hand, PCR primers are designed from the exact nucleotide sequences for a specific gene, therefore, such ambiguity can be eliminated. Variations at genomic sequence may also cause discrepancy between observed Ct values and basemen values, as apple rootstocks used here are known to be genetically distant from ‘Golden Delicious” from which the apple draft genome sequence was derived from [43]. It is also possible that the alternative splicing or allelic variations may exist between resistant and susceptible lines, which may result in the imperfect primer annealing sites. This speculation seems to be supported by Ct values from the “#58 Pu tissue type” and root tissue under abiotic stress, where Ct values were under 30 (#58 line is a resistant line). In short, all candidate genes demonstrated a medium-level expression in various root tissue samples as expected except MDP0000211280, and all have the unique amplicon and adequate PCR efficiency.

### Assessment of the expression stability measure (M) using geNorm

**Fig 2:**
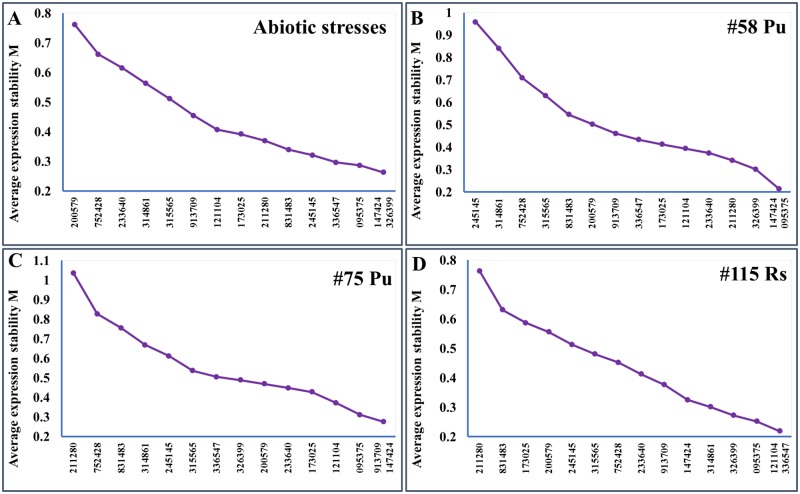
Gene expression stability (M) of candidate genes calculated by geNorm. (A) Abiotic stresses treated root tissues from cultivar ‘Gala’; (B) *P*. *ultimum* infected #58 roots; (C) *P*. *ultimum* infected #75 roots; (D) *R*. *solani* infected #115 roots. The least stable genes are on the left, while the most stable genes are on the right.

**Fig 3:**
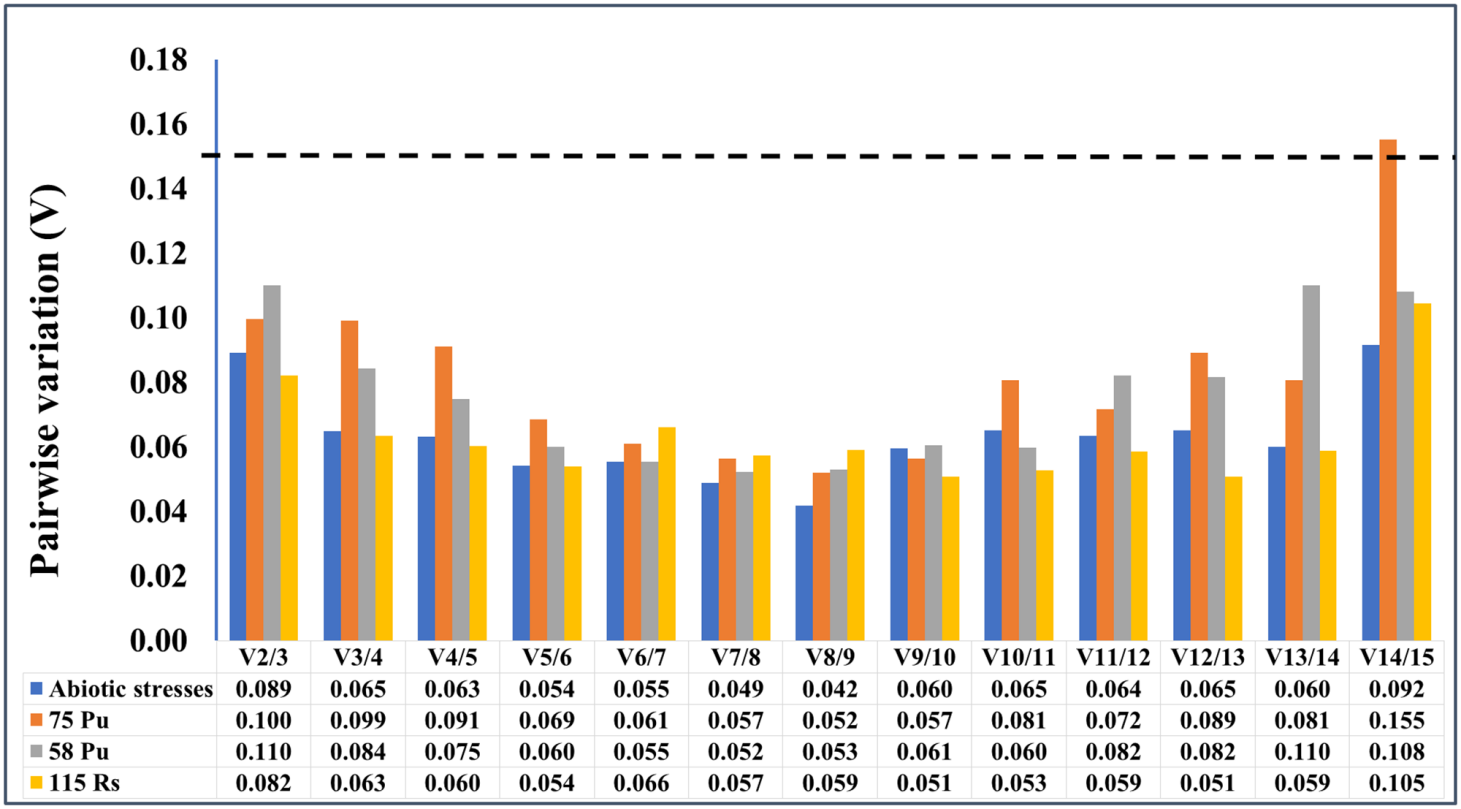
Determination of the optimal number of reference genes. The pairwise variation (Vn/Vn+1) was calculated between the normalization factors NFn and NFn+1 by geNorm. If the Vn/Vn+1 value is less than 0.15, there is no need to add an additional gene for normalization.

The raw Ct values themselves are simply the indicator of gene expression levels, which cannot represent the stability of candidate reference gene expression. The expression stability of these candidate reference genes was evaluated using geNorm algorithm [28]. The assessment of expression stability measure (M) is based on the average pairwise expression ratio between genes being studied until the lowest value is generated between the last two refence genes (Fig 2). Candidate gene with the lowest M value indicates the highest stability. All genes studied showed the acceptable expression stabilities, as their M values were all in accordance with the limit of <1.5 as suggested by geNorm. The two best-performing reference genes for each sample (genotype x treatment) were as follow: MDP0000147424 and MDP0000326399 for abiotic treatments (Fig 2A), MDP0000147424 and MDP0000095375 for “#58 Pu” (Fig 2B), MDP0000913709 and MDP0000147424 for “#75 Pu” (Fig 2C), and MDP0000121104 and MDP0000336547 for “#115 Rs” (Fig 2D). Notably, MDP0000147424 was the top-ranked in three out of four tissue types. In addition, the optimal number of reference genes required for a more reliable normalization was calculated by geNorm. Since the V_2/3_ values for all treatments were less than the cut-off value 0.15, the results based on geNorm analysis indicated that two reference genes are sufficient for PCR data normalizations (Fig 3).

### Integrated evaluation on expression stability

The expression data for all 15 candidate reference genes were further evaluated for their expression stability by other two methods: NormFinder [27] and BestKeeper [29]. NormFinder is an ANOVA based analysis, which integrates intra- and inter-group variations in assessing the expression stability [27]. NormFinder determined that MDP0000095375 from abiotic treatments, MDP0000233640 from “#75 Pu”, MDP0000147424 from “#58 Pu” and MDP0000095375 from “#115 Rs” as the most stable genes under these four conditions, respectively. As NormFinder takes into the account of compensation effect from the expression levels by two reference genes used, the best combinations of top-two reference genes calculated by NormFinder were: MDP0000095375 and MDP0000326399 for abiotic treatments, MDP0000200579 and MDP0000315565 for “#75 Pu”, MDP0000831483 and MDP0000147424 for “#58 Pu”, and MDP0000121104 and MDP0000095375 for “#115 Rs”. These results demonstrated that the best combination of two reference genes are not always the top-rated reference genes.

**Fig 4:**
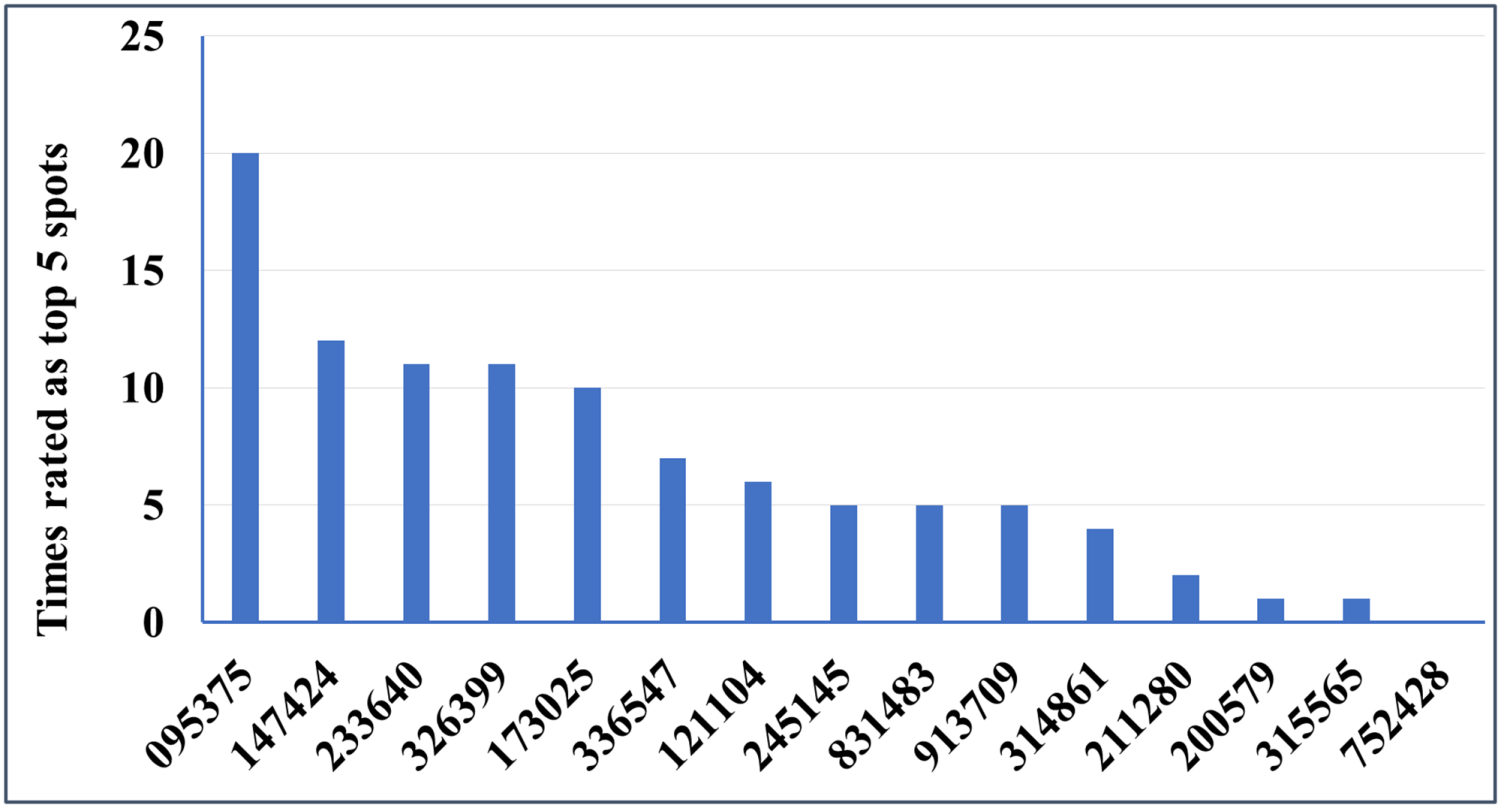
The frequency of appearance in top five spots by individual tested genes. Base on the ranking order by four different analytic methods and comprehensive ranking (see detail in Table 3), the values of Y axis indicate the times for an individual gene being ranked in the top five spots from all four tissue types used. The distribution of the top-rated candidate genes from evaluation by various methods were combined to indicate their overall suitability as the most reliable reference genes for gene expression analysis in apple root tissues.

BestKeeper is another commonly used normalization algorithm which is designed to select the most stable genes according to the standard deviation (SD) and coefficiency of variation (CV) of Ct values. BestKeeper confirmed that MDP0000245145, MDP0000095375, MDP0000831483 and MDP0000233640 were ranked in the highest positions for abiotic treatments, “#75 Pu”, “#58 Pu”, and “#115 Rs”, respectively. It is known that BestKeeper has a limitation where genes are likely to be ranked highly if they demonstrate co-ordinate regulation, even if they are not truly stable [44]. Integrated evaluation by RefFinder [39] combines the assessment on gene expression stability from all four methods, and ranks the overall suitability for these candidate reference genes (Table 3). Several genes are consistently being rated as the high stability of expression by geNorm analysis, NormFinder and BestKeeper such as MDP0000147424 and MDP0000095375. Base on the frequency of their appearances at top five spots by all four evaluation methods, the highly-rated candidate genes among all 15 selected genes were MDP0000095375, MDP0000147424, MDP0000233640, MDP0000326399 and MDP0000173025 (Fig 4).

**Table 3:** Stability ranking of 15 candidate reference genes of four treatments.

Tissue types and analytic methods	Ranking order (1 being the most stable, 15 being the least stable)														
	1	2	3	4	5	6	7	8	9	10	11	12	13	14	15
*abiotic treatments*															
Δ Ct	095375	326399	147424	245145	336547	173025	211280	831483	121104	913709	233640	315565	752428	314861	200579
geNorm	147424/326399		095375	245145	831483	336547	173025	211280	121104	913709	315565	314861	233640	752428	200579
NormFinder	095375	326399	147424	173025	245145	211280	336547	121104	831483	913709	233640	752428	315565	314861	200579
BestKeeper	245145	326399	095375	121104	211280	173025	831483	147424	336547	233640	913709	315565	314861	752428	200579
**Comprehensive**	**326399**	**095375**	**147424**	**245145**	**173025**	**211280**	**336547**	**831483**	**121104**	**913709**	**233640**	**315565**	**752428**	**314861**	**200579**
75 Pu															
Δ Ct	095375	233640	913709	147424	173025	200579	336547	121104	326399	315565	245145	314861	831483	752428	211280
geNorm	913709/147424		095375	121104	173025	233640	200579	326399	336547	315565	245145	314861	831483	752428	211280
NormFinder	233640	336547	095375	200579	913709	173025	326399	315565	147424	121104	245145	314861	831483	752428	211280
BestKeeper	095375	233640	173025	913709	315565	121104	200579	336547	147424	326399	245145	314861	831483	752428	211280
**Comprehensive**	**095375**	**233640**	**913709**	**147424**	**173025**	**200579**	**336547**	**121104**	**326399**	**315565**	**245145**	**314861**	**831483**	**752428**	**211280**
58 Pu															
Δ Ct	095375	147424	173025	233640	326399	121104	336547	913709	211280	831483	200579	315565	752428	314861	245145
geNorm	147424/095375		326399	211280	233640	121104	173025	336547	913709	200579	831483	315565	752428	314861	245145
NormFinder	095375	147424	173025	831483	233640	326399	336547	121104	913709	200579	211280	315565	752428	314861	245145
BestKeeper	831483	173025	095375	147424	233640	315565	200579	336547	913709	326399	121104	211280	752428	314861	245145
**Comprehensive**	**095375**	**147424**	**173025**	**831483**	**233640**	**326399**	**336547**	**121104**	**211280**	**913709**	**200579**	**315565**	**752428**	**314861**	**245145**
115 Rs															
Δ Ct	095375	121104	336547	326399	314861	147424	233640	913709	752428	315565	200579	173025	245145	831483	211280
geNorm	121104/336547		095375	326399	314861	147424	913709	233640	752428	315565	245145	200579	173025	831483	211280
NormFinder	095375	336547	121104	326399	314861	147424	233640	913709	200579	752428	173025	315565	245145	831483	211280
BestKeeper	233640	095375	831483	336547	314861	913709	121104	326399	147424	200579	315565	173025	752428	245145	211280
**Comprehensive**	**095375**	**336547**	**121104**	**233640**	**326399**	**314861**	**147424**	**913709**	**831483**	**752428**	**200579**	**315565**	**173025**	**245145**	**211280**

MDP: predicted gene model from the apple (*Malus* x *domestica*) genome sequences v3.0.a1 (https://www.rosaceae.org/analysis/162). The six-digit numbers represent individual apple gene models without the prefix of MDP0000 for conciseness.

As other noticed [17] and observed in this study, the best reference genes are likely specific to tissue types or experimental conditions. For example, while 245145 and 211280 were shown among the lowest rating among tissues under biotic stresses from infection by two pathogens, their rating under abiotic stresses condition were ranked at 4th or 6th spot, respectively. An actin gene (MDP0000752428), which has been previously selected based on its house-keeping function and been used for RT-qPCR data normalization in both fruit and root tissues (31); in this study, it was listed as the last place as determined by the number of times of being rated in the top five spots. On the other hand, the M value of MDP0000752428 by geNorm analysis is at an acceptable level of less than 1.5. Noticeably, four of the previously reported apple reference genes, which were primarily validated using fruit tissues, were also among the top-ranked reference genes suitable for normalizing RT-qPCR data from apple root tissues. Our results highlight the values and need for careful reference gene validation, even for specific tissue types.

### Testing the validated reference genes on two target genes

**Fig 5:**
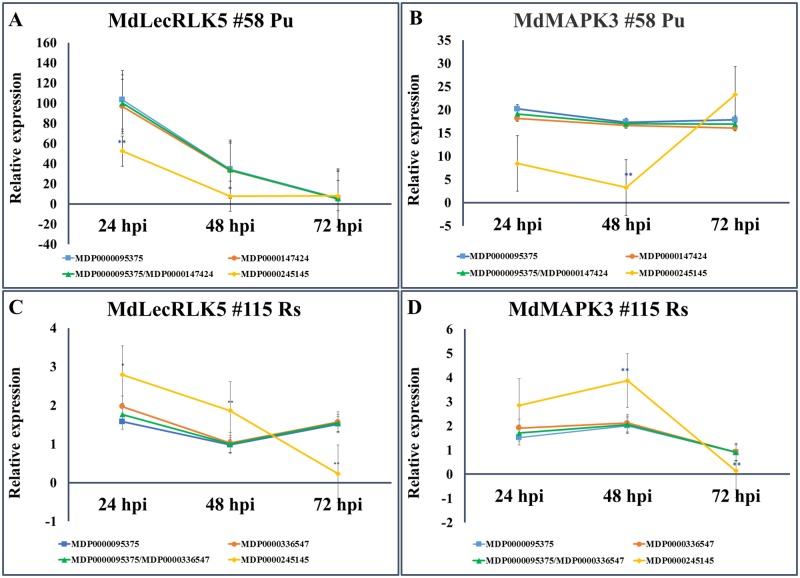
Normalized expression patterns of *MdLecRLK5* and *MdMAPK3* using selected reference genes. (A) *MdLecRLK5* and (B) *MdMAPK3* in “#58 Pu” root tissues at 24, 48 and 72 hpi; (C) *MdLecRLK5* and (D) *MdMAPK3* in “#115 Rs” root tissues at 24, 48 and 72 hpi. The error bars are standard errors. T-test statistics were generated by ANOVA among relative expression levels in the same sample. * P<0.05, ** P<0.01.

The expression data of two target genes, *MdLecRLK5* and *MdMAPK3*, were used to test the reliability of the validated reference genes from current study. As shown previously (Table 3), the best rated reference genes were MDP0000095375, MDP0000147424 and the least stable reference gene was MDP0000245145 from the tissue type of “#58 Pu”. When these two top-rated reference genes (MDP0000095375, MDP00000147424) were used for data normalization, either singular or combinational, no significant differences of expression levels were observed at each time point for both target genes (Fig 5A and 5B). However, when the least stable reference gene (MDP0000245145) was used for data normalization for both target genes, significant differences of expression levels were detected at some data points, comparing those data to normalized data by two top-rated reference genes (MDP0000095375, MDP0000147424). Similar results were observed in root tissue series of “#115 Rs” (Fig 5C and 5D). When the most stable reference genes (MDP0000095375 and MDP0000336547) (Fig 3C and 3D) were used for normalization of the expression data for these two target genes, no significant difference of relative expression levels for both target genes were observed, either singular or combinational of two reference genes were applied. On the other hand, when the least stable reference gene (MDP0000245145) was used for data normalization, the expression levels were significantly different from those normalized by the two top-rated reference genes at most time points.

The differences of normalized expression data for these two functional genes, using either the most or least stable reference genes, clearly demonstrated the impact of reference gene on the interpretation of gene expression dynamics. Although the expression patterns of these two target genes were not reversed or changed in most cases except one (Fig 5C), the relative expression levels were significantly affected due to the choices of different reference genes. Also, because MDP0000095375 was rated as the top performer in both tissue types used, all three reference genes (MDP0000095375, MDP0000147424 and MDP0000336547) should be equally suitable reference genes for normalizing gene expression data for at least both tissue types, or perhaps most root tissue types. Result from this study also seems to suggest that for those top-rated reference genes, normalization by a single gene is sufficient, as no significant differences were observed from using both reference genes singularly or in combination. The data indicated that reference gene validation is an important step for accurate and reliable interpretation of gene expression patterns in any specific tissue types. From this study, a panel of reliable reference genes were identified for RT-qPCR data normalization in apple root tissues.

## Conclusions

Apple root health is fundamental for tree productivity and orchard viability, but apple roots are the less studied subject for quantitative gene expression analysis as they are compared with apple fruit or leaf tissues. Reference genes which are specifically suitable for RT-qPCR data normalization have not been carefully validated in apple root tissues under different treatments. Taking the advantages of a comprehensive RNA-seq based transcriptome dataset for apple root response to infection by *P*. *ultimum*, a set of candidate genes exhibiting low variance of expression were subjected to a systematic analysis of expression stability by several methods. The expression of these candidate reference genes was carefully examined using apple root samples of a variety of genotypes and under abiotic stresses or pathogen infection. Although the ranking of the stable reference genes can be variable across tissue types or analytic methods, several of the tested genes consistently ranked at the top five spots. When two of the top-rated reference genes were used to normalize the expression data of selected target genes, either singularly or in combination, no significant differences of normalized data were found at all data points. On the other hand, between normalized data by two most stable reference genes and those normalized by the least stable reference gene significant differences of expression level were observed at most data points. Our data indicated that, for these carefully validated reference genes, single reference gene is sufficient for reliable normalization of the quantitative gene expression data in apple roots. In the meantime, the most suitable reference genes can be variable depending on root tissue types or different treatments. The validation of this panel of reference genes is a valuable contribution for RT-qPCR analysis in apple root tissues of different genotypes, under various abiotic stresses and during pathogenesis.

### Disclosure

Mention of tradenames or commercial products in this publication is solely for providing specific information and does not imply recommendation or endorsement by the U.S. Department of Agriculture.
